# Supplementary material for: Achieving Minimum Standards for Infection Prevention and Control in Sierra Leone: Urgent Need for a Quantum Leap in Progress in the COVID-19 Era!
Source: Int J Environ Res Public Health. 2022 May 6;19(9):5642. doi: 10.3390/ijerph19095642 (PMC9102022; doi:10.3390/ijerph19095642)
Supplement: Supplementary file 1 [file ijerph-19-05642-s001.zip › Tables S1 and S2.pdf]

Supplementary Table S1: Percentage scores of the core components of IPCAF in four regional hospitals of Sierra Leone during 2019 (pre-COVID-19) and 2021 (COVID-19 era) assessment.

**IPC Component***	**RH1**		**RH2**		**RH3**		**RH4**	
	**2019**	**2021**	**2019**	**2021**	**2019**	**2021**	**2019**	**2021**
	**Score (%)**	**Score (%)**	**Score (%)**	**Score (%)**	**Score (%)**	**Score (%)**	**Score (%)**	**Score (%)**
i. IPC programme	43	50	40	50	43	45	40	48
ii. IPC guidelines	65	68	65	75	65	75	65	68
iii. IPC education and training	70	85	65	85	50	75	25	80
iv. HAI surveillance	3	43	3	43	5	28	0	45
v. Multimodal strategies	40	45	35	45	35	45	45	45
vi. Monitoring/audits of IPC practices and feedback	30	63	30	50	20	48	10	65
vii. Workload, staffing and bed occupancy	25	30	25	35	25	30	20	25
viii. Built environment, materials and equipment for IPC	49	61	46	63	43	49	41	54
**Cumulative score**	324	444	309	445	285	394	246	429
**Cumulative score (%)**	**40**	**55**	**39**	**56**	**36**	**49**	**31**	**54**

Abbreviation: IPC= Infection, Prevention and Control; HAI= Healthcare Associated Infection

* Maximum score for each component is 100 and for the cumulative it is 800

Percentages are calculated relative to the maximum score for the component

Grade: IPC performance in each component will be graded based on the obtained percentage: i) inadequate (0%-25%) ii) basic (25.1%-50%) iii) intermediate (50.1%-75%) and iv) advanced (75.1%-100%)

Supplementary Table S2: Percentage scores of the core components of IPCAF in eight district hospitals of Sierra Leone during 2019 (pre-COVID-19) and 2021 (COVID-19 era) assessment.

**IPC Component***	**DH 1**		**DH 2**		**DH 3**		**DH 4**		**DH 5**		**DH 6**		**DH 7**		**DH 8**	
	**2019**	**2021**	**2019**	**2021**	**2019**	**2021**	**2019**	**2021**	**2019**	**2021**	**2019**	**2021**	**2019**	**2021**	**2019**	**2021**
i. IPC programme	30	40	40	40	38	28	25	48	40	50	40	28	33	40	33	25
ii. IPC guidelines	65	68	65	68	65	68	65	68	65	75	65	68	65	68	65	65
iii. IPC education and training	60	60	60	65	30	80	65	80	45	80	15	65	55	80	55	70
iv. HAI surveillance	0	45	0	45	0	45	0	50	10	5	0	45	0	50	0	10
v. Multimodal strategies	55	45	55	45	35	45	40	45	35	45	45	45	35	45	35	45
vi. Monitoring/audits of IPC practices and feedback	43	58	45	53	50	58	43	50	25	58	20	48	28	53	28	30
vii. Workload, staffing and bed occupancy	25	40	25	25	20	25	25	25	15	30	25	45	25	25	25	30
viii. Built environment, materials and equipment for IPC	43	50	49	51	45	49	58	55	58	51	50	55	36	50	36	51
**Cumulative score**	**320**	**405**	**339**	**391**	**283**	**396**	**320**	**420**	**293**	**394**	**260**	**398**	**276**	**410**	**276**	**326**
**Cumulative %**	**40**	**51**	**42**	**49**	**35**	**50**	**40**	**53**	**37**	**49**	**33**	**50**	**35**	**51**	**35**	**41**

Abbreviation: IPC= Infection, Prevention and Control; HAI= Healthcare Associated Infection

* Maximum score for each component is 100 and for the cumulative it is 800

Percentages are calculated relative to the maximum score for the component

$ The median scores from the eight facilities

Grade: IPC performance in each component will be graded based on the obtained percentage: i) inadequate (0%-25%) ii) basic (25.1%-50%) iii) intermediate (50.1%-75%) and iv) advanced (75.1%-100%)
